# Supplementary material for: Factors associated with unfavorable treatment outcomes in patients with rifampicin-resistant tuberculosis in Colombia 2013–2015: A retrospective cohort study
Source: PLoS One. 2021 Apr 14;16(4):e0249565. doi: 10.1371/journal.pone.0249565 (PMC8046199; doi:10.1371/journal.pone.0249565)
Supplement: S1 Table — (DOCX) [file pone.0249565.s001.docx]

**S1 Table. Sociodemographic, clinical and laboratory characteristics of included and excluded patients in the MDR/RR-TB cohort in Colombia, 2013-2015**

|  | **included** | | **excluded** | | **P*** |
| --- | --- | --- | --- | --- | --- |
|  | **n** | **%** | **n** | **%** |  |
| Age (years) |  |  |  |  | 0.947 |
| ˂20 | 39 | 86,7 | 6 | 13,3 |  |
| 20 to 39 | 186 | 85,3 | 32 | 14,7 |  |
| 40 to 59 | 168 | 87,0 | 25 | 13,0 |  |
| ≥60 | 48 | 87,3 | 7 | 12,7 |  |
| Sex |  |  |  |  | 0.058 |
| Male | 285 | 88,5 | 37 | 11,5 |  |
| Female | 156 | 82,5 | 33 | 17,5 |  |
| Self-reported ethnicity |  |  |  |  | 0.245 |
| Indigenous | 12 | 85,7 | 2 | 14,3 |  |
| Afro-Colombians | 72 | 92,3 | 6 | 7,7 |  |
| Mestizos | 357 | 85,2 | 62 | 14,8 |  |
| Tuberculosis |  |  |  |  | 0.803 |
| Pulmonary | 407 | 86,4 | 64 | 13,6 |  |
| Extrapulmonary | 34 | 85,0 | 6 | 15,0 |  |
| Health regime |  |  |  |  | 0.080 |
| Subsidized | 299 | 88,2 | 40 | 11,8 |  |
| Contributive | 142 | 82,6 | 30 | 17,4 |  |
| Level of care |  |  |  |  | 0.377 |
| Primary | 309 | 85,1 | 54 | 14,9 |  |
| Secondary and tertiary | 113 | 88,3 | 15 | 11,7 |  |
| Method used for diagnosis of resistance |  |  |  |  | 0.140 |
| Bactec MGIT^b^ | 194 | 86,6 | 30 | 13,4 |  |
| Lipa^c^ | 120 | 87,6 | 17 | 12,4 |  |
| Real time PCR^d^ | 82 | 80,4 | 20 | 19,6 |  |
| LJ^e^ Proportions | 45 | 93,8 | 3 | 6,3 |  |
| Type of resistance |  |  |  |  | 0.948 |
| XDR^f^ | 14 | 87,5 | 2 | 12,5 |  |
| MDR^g^ | 313 | 86,0 | 51 | 14,0 |  |
| Monoresistance to RMP^h^ | 114 | 87,0 | 17 | 13,0 |  |

^a^p-value calculated by the Chi-square test; ^b^ mycobacterial growth indicator tube; ^c^line probe assay; ^d^ polymerase chain reaction; ^e^ Löwenstein-Jensen; ^f^ extensively drug resistant; ^g^ multidrug resistant, ^h^ rifampin
