# Supplementary material for: Factors associated with unfavorable treatment outcomes in patients with rifampicin-resistant tuberculosis in Colombia 2013–2015: A retrospective cohort study
Source: PLoS One. 2021 Apr 14;16(4):e0249565. doi: 10.1371/journal.pone.0249565 (PMC8046199; doi:10.1371/journal.pone.0249565)
Supplement: Attachment — Submitted filename: Peer Review and Cover Letter.pdf [file pone.0249565.s002.pdf]

28th July 2020

Dear Dr Wingfield,

Thank you for the opportunity to review the attached paper entitled ‘Factors associated with unfavourable treatment outcomes in patients with rifampicin-resistant tuberculosis in Colombia’.

I enjoyed reviewing the paper and recognise its important contribution to the social epidemiology of drug resistant tuberculosis in Colombia, and in greater context in Latin America. This paper is a national retrospective cohort study using clinical registry data, which evaluates the outcomes of patients who started treatment for rifampicin resistant tuberculosis between January 2013 and December 2015. It contributes new insights into the socio-demographic and health characteristics of people undergoing treatment for rifampicin resistant tuberculosis in Colombia, and identifies risk factors for unfavourable treatment outcome in this group.

This paper may also provide some indication of inequalities within the Colombian health care system for patients undergoing treatment for rifampicin resistant tuberculosis, with findings that individuals in receipt of subsidised health care are independently three times as likely to experience unfavourable outcomes than their counterparts. However, these findings are limited by the study sample size, lack of household level data, and lack of adjustment for other important confounding variables, such as education and socioeconomic status, and the study conclusions should reflect this.

I would therefore like to recommend that this paper would benefit from revision prior to publication, and have structured my suggestions by major and minor revisions in the attached review.

Kind regards,

Dr Louisa Chenciner BMedSci BMBS MSc (Hons)

Major Revisions

- **Statistical Analysis in Methodology**
  - Please mention clearly that you first performed bivariate analysis, and whether thereafter included variables with p value < 0.05
  - Please clarify whether, and which, variables were included or excluded a priori from the multivariate analysis to clarify the model building process
  - Please specify which confounders were included in the analysis here, and which (if any) registry variables were excluded
- **Descriptive Analysis**
  - Were any other variables excluded due to missing data? Would be helpful to specify % of missing data here
  - Figure 1: Can you clarify why you decided to exclude 396 individuals with INH resistant TB? Did you perform a separate analysis which included this group to see how outcomes differed?
  - Table 1: How and why did you choose to disaggregate age into <20, 20 to 39, 40 to 59 and over 60 – it appears the majority are in the 20-59 bracket and valuable information may be lost by grouping into such large categories. Did you perform a sensitivity analysis using smaller age intervals?
  - There are only 16 patients with XDR TB; are you convinced this sample is large enough to make meaningful conclusions about the rate of unfavourable treatment outcomes in this group?
  - Please rationalise why you have included method of diagnosis in your analysis, how do you anticipate this would be associated with treatment outcome?
- **Results: Treatment outcomes and factors associated with Unfavourable outcomes**
  - Consider using primary healthcare as reference group in this analysis; it seems unsurprising here that primary healthcare is a 'protective factor' as surely this variable is just a proxy for patients being 'less sick'. I would be more interested to see whether those receiving secondary/tertiary level care had better or worse outcomes – does being in a tertiary centre result in more specialised care or are patients sicker?
- **Discussion**
  - *'The strong association between health regime and TB treatment outcomes in Colombia is relevant, as individuals affiliated with the government-subsidized health regime, i.e., the poorest and most vulnerable population, had a higher probability of presenting unfavourable treatments'* and *'The conditions of poverty and vulnerability of the population affiliated with the subsidized regime may be associated with different barriers accessing basic and health services'* - This feels like a leap; given your model does not include household income, education level, living conditions – isn't qualifying for subsidised healthcare a proxy for socioeconomic deprivation in this analysis? It may be difficult to confirm whether the discrepancy in treatment outcome for this group relates to barriers to access, quality of care, or other social factors for unfavourable outcome. Consider rephrasing and discussing these limitations more fully.
  - *'Therefore we carry out a careful adjustment of possible confounding factors, seeking to reduce biases'*- what kind of bias did you seek to reduce, and

what other confounders should have been included? Please explain how your results may have been affected (e.g. over/underestimation of odds ratios) by these confounders being omitted

  - I think you need to address other limitations to this study e.g. limited sample size, lack of household level characteristics, lack of information on the preceding treatment regimen, and other confounding variables e.g. markers of socioeconomic deprivation and education which are not recorded in this registry, and limited information provided about ethnicity and race. Might be helpful to consider who is not captured by this registry

- **Conclusions**
  - Try to align the conclusions more closely with your results and discussion

<u>Minor Revisions</u>

- **Title**
  - Could be tweaked to correspond directly to its contents, there is some interchangeable reference between MDR-TB, DR-TB, XDR-TB, RR-TB and RMP. Would also be good to specify that it is a retrospective cohort study and registry based in the title
- **Abstract**
  - Background: could you clarify what you mean by ‘exerts great pressure on the complex Colombian health system’, e.g. interesting to know whether you are referencing treatment costs, lengthy treatment, inadequate infrastructure?
  - Methodology: If able please specify the study inclusion criteria, and that the study uses registry level data in the abstract. Are you including those who have received previous treatment?
  - Results: Language used in the results is inconsistent with the methodology in the abstract, would be helpful to review the sentences ‘511 patients who started treatment for MDR/RR-TB’ and ‘A total of 511 patients were diagnosed with RR-TB in Colombia’. Suggest reviewing the language ‘affiliated with the subsidized health regime’, for example could state ‘individuals who qualified for subsidised health care services’
  - Consider making it clearer in the abstract whether you included all patients with DR-TB in your regression model, or whether you performed sub-group analysis comparing MDR-TB, RMP and XDR-TB
  - Suggest reviewing key words to ensure they relate more closely to your paper, e.g. consider dropping ‘associated factors’

- **Introduction**
  - Suggest rephrasing ‘these conditions represent public health problems around the world’, and relating more directly to the challenge that DR-TB poses
  - Line 61 – are these definitely marked ‘improvements’?
  - Suggest potential restructuring, and position paragraph 3 first – strengthen argument as to why this is a particular public health concern in Colombia
  - Please ensure that in-text citations are inside of the sentence, e.g. before full stop.

- *'In 2018, 205 67 cases were notified out of an estimated 580, for a detection rate of 35.3%, similar to 68 the detection rate of 32% reported worldwide'* – please clarify which cases you are referring to; it sounds like you suspect there is underreporting of DR-TB in Colombia?
        - Several references to 'pressure' and the 'complex Colombian health System' – please clarify in which respect, e.g. are you referencing lack of resources, infrastructure, workforce?
        - Suggest maintaining consistent language, e.g. stick with treatment, not mentioning pharmacotherapy in the study objective
- **Methods**
    - **Study Site**
        - You may be able to better summarise or condense the provision of healthcare in Colombia by using a figure or schematic
        - Please confirm whether subsidised healthcare includes additional support, e.g. social protection measures
        - Suggest paragraph 1 e.g. line 89-93 could be better summarised and more clearly convey the importance of understanding DR-TB in this study setting
        - Could be helpful to mention earlier that the direct costs of treatment are 'free' at point of care, but there are hidden costs
        - Also please confirm that TB is a notifiable disease in Colombia
    - **Study Type and Population**
        - Please consider specifying whether the dataset was anonymised or de-identified
        - Helpful to provide exact dates of study time frame, given you later state it might have been too early for treatment outcomes to be recorded for those where its treatment outcome was missing
        - Please confirm when treatment outcome was recorded (e.g. time to follow-up), and how
        - Notification and diagnosis are used interchangeably, helpful to stick to one term only as these can mean slightly different things
    - **Statistical Analysis in Methodology**
        - Reconsider the language 'analyze the interaction', this does not really reflect the statistical analysis reported in the results – unless you did examine interaction terms? Otherwise suggest 'to estimate the association between…'
        - Please specify which confounders were included in the analysis here, and which (if any) registry variables were excluded
    - **Definition of Terms**
        - Suggest restructuring to first define outcome variables e.g. 'favourable' and 'unfavourable' and thereafter how DR-TB is defined in Colombia. It is slightly misleading to first define the clinical rationale needed to 'suspect' DR-TB, followed by criteria for the diagnosis of DR-TB – as not totally clear on how DR-TB was defined in your study inclusion criteria
        - Specify what you mean by the 'Colombian National Policy'

- **Results**
  - **Descriptive Analysis**
    - Figure 2: Is it necessary to show rates of both favourable and unfavourable treatment outcomes on the same graph? Please include y axis for scale. Consider excluding this figure, I am not sure that it helps to answer the study objective.
    - What is the derivation of 'afro-descendant'? Perhaps consider changing to Afro-Colombian. Who comprises the 'other' group – it seems this is the 'majority' – How has ethnicity been defined here?
    - Reconsider phrasing 'first', 'second' and 'third' care as 'primary', 'secondary' and 'tertiary'
    - On line 211, please reconsider 'were affiliated with the subsidized social security and health regime'. Does the subsidised health care also include social protection and other welfare benefits? This contradicts the earlier definition
  - **Treatment outcomes and factors associated with Unfavourable outcomes**
    - Describing 'the treatment success rate with respect to age showed an inversely proportional trend' seems like a bit of a leap; could be worth rephrasing, and focussing on the needs of this group more in your discussion
    - Table 2: Given you have performed a binomial logistic regression e.g. with a dichotomous outcome variable not multinomial logistic regression, it feels misleading to show success vs. abandonment vs. failure vs. death in Table 2, consider formatting Table 2 with favourable vs. unfavourable outcomes. Please amend 'IC95%' to '95% CI'. Consider rearranging the reference group to first sub-group for every covariate of interest. Please avoid using commas as decimal points. Please include 'Ref.' in your figure legend. Do you think that the 'self-identified ethnicity' variable is limited in its use here?

- **Discussion**
    - Suggest condensing paragraph one and focussing more on your own results and placing them into context, e.g. in the region and then globally
    - *'Notably, the diagnosis and treatment of MDR/RR-TB in Colombia is free for all patients regardless of health regime affiliation'* – so actually we are seeing the impact of hidden costs of treatment here? Worth clarifying what is and is not included in your treatment if you are in the subsidised group. Would be helpful to include some background as to catastrophic health spending in Colombia for patients with TB, or more broadly (https://www.researchgate.net/publication/50228851_Determining_factors_of_catastrophic_health_spending_in_Bogota_Colombia)
    - *'At the national level, incidence and mortality rates, adjusted for age and sex, were higher for the subsidized regime in 37 events of public health interest'* – please clarify what these 37 events are? And how meaningful is this if incidence and mortality has only been adjusted by age and sex.
    - *'Other countries such as Nigeria also documented a higher rate of successful TB treatment among patients treated in the private sector'* – however being treated in a public healthcare facility was not independently associated with unfavourable treatment outcome in your multivariate analysis– so I think

this comparison may be limited. Are there any other comparable studies in South America?

- *'The care received at first-level HCIs (health centers) was a protective factor for unfavorable outcomes'*- Again this did not persist on adjustment in your multivariate model, and it could be worth exploring this further in your discussion
- Please consider using sub-headings in the discussion to clearly structure this section

- **Conclusions**
  - Please consider rephrasing *'This high rate of unfavourable treatment outcomes was associated with affiliation with the subsidized health system regime and age ≥ 60 years.'* - e.g. could be adapted to: Those who qualified for subsided health care or who were aged over 60 years were independently more likely to experience unfavourable treatment outcomes
