# Supplementary material for: Factors associated with unfavorable treatment outcomes in patients with rifampicin-resistant tuberculosis in Colombia 2013–2015: A retrospective cohort study
Source: PLoS One. 2021 Apr 14;16(4):e0249565. doi: 10.1371/journal.pone.0249565 (PMC8046199; doi:10.1371/journal.pone.0249565)
Supplement: Attachment — Submitted filename: Response to Reviewers.docx.pdf [file pone.0249565.s003.pdf]

Dr. Tom E. Wingfield
Academic Editor
PLOS ONE Response to Reviewers

Ref: PONE-D-20-18648
Factors associated with unfavorable treatment outcomes in patients with rifampicin-resistant tuberculosis in Colombia

Dear Dr. Tom E. Wingfield

We then respond to questions and suggestions from the editor and reviewers. We look forward to meeting the requirements and expectations of PLOS ONE. We are available for further questions or suggestions about this work

Journal requirements:

1. Please ensure that your manuscript meets PLOS ONE's style requirements, including those for file naming. The PLOS ONE style templates can be found at

https://journals.plos.org/plosone/s/file?id=wjVg/PLOSOne_formatting_sample_main_body.pdf and

https://journals.plos.org/plosone/s/file?id=ba62/PLOSOne_formatting_sample_title_authors_affiliations.pdf

OK.

Please can you systematically address the reviewers' comments paying special attention to:
1) Enhancing the literature review and introduction with relation to social

determinants of TB and their further association with adverse TB treatment outcomes
2) Clarify some parts of the methods including: inclusion and exclusion criteria; evidence informing inclusion of independent variables into your regression model (e.g. method of diagnosis); and methods used to arrive at your adjusted model
3) Improvements to the written text and flow
4) Adjustments to the Tables and Figures (see Reviewer 2's comments) to ensure that only the most relevant and applicable information is included and the tables and figures are supportive of the main study objectives.

We will systematically address the reviewers' comments paying special attention to these four items

Reviewer #1

Major Revisions

1. **Please mention clearly that you first performed bivariate analysis, and whether thereafter included variables with p value < 0.05**
   Line: 130 to 134 “Bivariate analysis: A logistic regression model was used to analyze the interaction of the exposure variables (age, sex, ethnicity, site of TB, health insurance scheme, treating HCI, level of care, method used for diagnosis and type of resistance) with the response variable (treatment outcome). Variables with p-values < 0.05 in the bivariate analysis were considered for the multivariate analysis”

2. **Please clarify whether, and which, variables were included or excluded a priori from the multivariate analysis to clarify the model building process**
   Line: 133 to 135 “Variables with p-values < 0.05 in the bivariate analysis were considered for the multivariate analysis. We did not include or exclude a priori variables from the multivariate analysis”.

3. **Please specify which confounders were included in the analysis here, and which (if any) registry variables were excluded.**
   A possible confounding variable included in the analysis is the health institution (public or private). We do not exclude registry variables.

4. **Were any other variables excluded due to missing data? Would be helpful to specify % of missing data here**
   Other variables were not excluded, the percentage of data lost was 13.7%
   Line: 194 “and 70 (13,7%) patients were excluded because treatment outcomes was unknown”

5. **Figure 1: Can you clarify why you decided to exclude 396 individuals with INH resistant TB? Did you perform a separate analysis which included this group to see how outcomes differed?**
   Only rifampicin-resistant patients were included in this study. Because in Colombia the treatment is different: those resistant to rifampicin receive second-line treatment that includes fluoroquinolones. Patients with isionezide resistance receive R E Z daily for 9 months.
   A separate analysis was performed that is not included in this publication, where it was observed that the results of treatment in patients with isoniazid resistance are very similar to those with drug-sensitive tuberculosis.

6. **Table 1: How and why did you choose to disaggregate age into <20, 20 to 39, 40 to 59 and over 60 – it appears the majority are in the 20-59 bracket and valuable information may be lost by grouping into such large categories. Did you perform a sensitivity analysis using smaller age intervals?**
   We arrived at this age categorization, after conducting sensitivity analysis, desegregating the variables at intervals of every 5 years and every 10 years.
   In this cohort, we noted that there were few patients younger than 20 years and older than 60 years, and in each patient the results of the treatment behaved

differently. While most of the patients were between 20 and 59 years old. This group was disaggregated into 2 groups almost equal in number of patients, trying to create comparable groups without losing much information.

7. **There are only 16 patients with XDR TB; are you convinced this sample is large enough to make meaningful conclusions about the rate of unfavourable treatment outcomes in this group?**
   16 patients is a small n to draw meaningful conclusions, and this could be a devil in this study. however, in Colombia, an average of 5 XDR cases are diagnosed and reported per year. and 16 patients are the total of patients diagnosed and reported with XDR in the 3 years of study. Therefore, studies that include long periods are recommended, because the incidence of XDR is not very high in Colombia.

8. **Please rationalise why you have included method of diagnosis in your analysis, how do you anticipate this would be associated with treatment outcome?**
   The diagnostic method of rifampicin resistance was included for a better description of the dynamics of the diagnosis and treatment of RR TB in Colombia; we did not expect it to influence the treatment results. Which was corroborated in the bivariate analysis and did not enter the multivariate analysis.
9. **Consider using primary healthcare as reference group in this analysis**; it seems unsurprising here that primary healthcare is a ‘protective factor’ as surely this variable is just a proxy for patients being ‘less sick’. I would be more interested to see whether those receiving secondary/tertiary level care had better or worse outcomes – does being in a tertiary centre result in more specialised care or are patients sicker?
   I agree that the best treatment results among patients seen in the first level of care may be related to the severity of the disease.

We consider using first-level care as a reference. We found that being seen at the second or third level could be associated with unfavorable results; however, this association was not significant.

Line 257 to 258 "In turn, being seen in a second or third level HCIs was associated with unfavorable results, although the association was not significant (ORc = 1.6; 95% CI: 1.0 - 2.5)"

10. **'The strong association between health regime and TB treatment outcomes in Colombia is relevant, as individuals affiliated with the government-subsidized health regime, i.e., the poorest and most vulnerable population, had a higher probability of presenting unfavourable treatments'**

    Line 274 to 277 "The association between the health regimen and the results of TB treatment in Colombia is relevant, since individuals affiliated with the government-subsidized health regimen, which are people with low economic resources, without the ability to pay contributions to the health system, were more likely to present unfavorable treatment results"

11. **and 'The conditions of poverty and vulnerability of the population affiliated with the subsidized regime may be associated with different barriers accessing basic and health services' - This feels like a leap; given your model does not include household income, education level, living conditions – isn't qualifying for subsidised healthcare a proxy for socioeconomic deprivation in this analysis? It may be difficult to confirm whether the discrepancy in treatment outcome for this group relates to barriers to access, quality of care, or other social factors for unfavourable outcome. Consider rephrasing and discussing these limitations more fully.**

    Line 285 to 290 "Establishing why the population affiliated with the subsidized health regime is more likely to have unfavorable TB treatment outcomes is beyond the scope of this study. Since we do not have enough socioeconomic variables such as family income to analyze in this cohort. Where previous

studies have suggested that economic barriers, including transportation, medication, and examination costs, or geographic barriers, cause these individuals to have limited access to health care".

12. **'Therefore we carry out a careful adjustment of possible confounding factors, seeking to reduce biases'- what kind of bias did you seek to reduce, and what other confounders should have been included? Please explain how your results may have been affected (e.g. over/underestimation of odds ratios) by these confounders being omitted**
Line 322 to 331 "so we carried out a multivariate analysis that included all the factors that were associated with unfavorable treatment outcomes in the bivariate analysis, seeking to highlight the factors that actually influence TB treatment outcomes and to rule out confounding factors. However we feel limited the possibility of studying other factors that could influence the outcomes of treatment for MDR/RR-TB, such as coinfection with HIV, comorbidities such as diabetes and habits such as alcohol, tobacco, psychoactive substance use and socioeconomic factors such as family income, as these variables were not considered in the data provided. The absence of these variables could lead to an overestimation of the association between health status and TB treatment outcomes"

13. **Try to align the conclusions more closely with your results and discussion**
Line 346 to 348 "Patients affiliated to the subsidized health regime were 3 times more likely to present unfavorable results than those affiliated to the contributory health regime, and age over 60 was also associated with unfavorable results"

Minor Revisions

- Title

1. Could be tweaked to correspond directly to its contents, there is some interchangeable reference between MDR-TB, DR-TB, XDR-TB, RR-TB and RMP. Would also be good to specify that it is a retrospective cohort study and registry based in the title

"Factors associated with unfavorable treatment outcomes in patients with rifampicin-resistant tuberculosis in Colombia 2013 - 2015 a retrospective cohort"

- Abstract

2. Background: could you clarify what you mean by 'exerts great pressure on the complex Colombian health system', e.g. interesting to know whether you are referencing treatment costs, lengthy treatment, inadequate infrastructure?

Line 80 to 82 "MDR/RR-TB requires prolonged and expensive treatment, which is difficult to sustain in a Colombian health system that requires the joint action of different actors to provide health services".

3. Methodology: If able please specify the study inclusion criteria, and that the study uses registry level data in the abstract. Are you including those who have received previous treatment?

A retrospective cohort study was conducted including all patients who initiated treatment for MDR/RR-TB between January 2013 and December 2015 in Colombia, who were registered and followed up by the national TB control program.

4. Results: Language used in the results is inconsistent with the methodology in the abstract, would be helpful to review the sentences '511 patients who started treatment for MDR/RR-TB' and 'A total of 511 patients were diagnosed with RR-TB in Colombia'. Suggest reviewing the language

‘affiliated with the subsidized health regime’, for example could state ‘individuals who qualified for subsidised health care services’

Line 40 to 41 “a total of 511 patients with MDR/RR-TB were registered and followed up by the national TB control program in Colombia”

“affiliated with the subsidized health regime” we consider that it is the term that best describes the characteristic

5. Consider making it clearer in the abstract whether you included all patients with DR-TB in your regression model, or whether you performed sub-group analysis comparing MDR-TB, RMP and XDR-TB

Line 48 to 52 “The 511 MDR/RR-TB patients were included in the bivariate and multivariate analysis, identifying that the age ≥ 60 years (crude odds ratio (ORc) = 2.4, 95% CI 1.1 – 5.8; adjusted odds ratio (ORa) = 2.7, 95% CI 1.1 – 6.8) and affiliation with the subsidized health regime (ORc = 3.6, 95% CI 2.3 – 5.6; ORa = 3.4, 95% CI 2.0 – 6.0) were associated with unfavorable treatment outcomes”.

6. Suggest reviewing key words to ensure they relate more closely to your paper, e.g. consider dropping ‘associated factors’

Line 59 to 60 “Tuberculosis, MDR/RR-TB, MDR-TB, treatment outcomes, unfavorable treatment outcomes”

Introduction

7. Suggest rephrasing ‘these conditions represent public health problems around the world’, and relating more directly to the challenge that DR-TB poses

Line 66 to 69 “These conditions are generally the consequence of social and political decisions that lead to inadequate compliance with the DOTs strategy (strictly supervised shortened treatment) by government entities,

sociodemographic barriers that prevent access to medicines (e.g. living in rural areas, not having health insurance)”

8. Line 61 – are these definitely marked ‘improvements’?

Line 74 to 76 “Although more people underwent treatment in 2018, they accounted for only 32% of the estimated incidence, and the treatment success rate was 56% for MDR/RR-TB and 39% for XDR-TB”

9. Please ensure that in-text citations are inside of the sentence, e.g. before full stop.

We make sure we put the points in the right place

10. ‘In 2018, 205 67 cases were notified out of an estimated 580, for a detection rate of 35.3%, similar to 68 the detection rate of 32% reported worldwide’ – please clarify which cases you are referring to; it sounds like you suspect there is underreporting of DR-TB in Colombia?

Line 82 to 83 “In 2018, 205 cases of MDR/RR-TB were notified out of an estimated 580”

We refer to cases of MDR/RR - TB.

We really believe that there is underreporting of RR-TB cases, but it is more worrying to think that RR-TB cases are not detected in a timely manner. However, we have no evidence to support this statement.

11. Several references to ‘pressure’ and the ‘complex Colombian health System’ – please clarify in which respect, e.g. are you referencing lack of resources, infrastructure, workforce? Suggest maintaining consistent language, e.g. stick with treatment, not mentioning pharmacotherapy in the study objective

The word Pressure was reformulated in the text and "complex health system" refers to the fact that the Colombian health system has three important actors, the government as a control entity, the companies that administer health services and the companies that provide health services. for a person to receive health care it is necessary that these three actors converge in favor of the individual and thus guarantee the provision of health services. For better understanding, this phrase was also substituted in the text.

Methods

Study Site

12. You may be able to better summarise or condense the provision of healthcare in Colombia by using a figure or schematic

We believe it is possible; however, the authors prefer to explain it in the text.

13. Please confirm whether subsidised healthcare includes additional support, e.g. social protection measures

Line 120 to 122 “However, people who qualified for subsidized health care services are not necessarily beneficiaries of social protection programs”

14. Suggest paragraph 1 e.g. line 89-93 could be better summarised and more clearly convey the importance of understanding DR-TB in this study setting

Line 111 to 113 “In this country of marked inequality, the social security system in health presents difficulties in responding to the economic and social demands involved in the treatment of MDR/RR-TB”.

15. Could be helpful to mention earlier that the direct costs of treatment are ‘free’ at point of care, but there are hidden costs. Also please confirm that TB is a notifiable disease in Colombia.

Line 136 to 138 “In summary, in Colombia TB is a disease of public health interest. Is of obligatory notification, its diagnosis, treatment and follow-up are covered by public resources”.

Study Type and Population

16. Please consider specifying whether the dataset was anonymised or de-identified

The dataset was anonymised

17. Helpful to provide exact dates of study time frame, given you later state it might have been too early for treatment outcomes to be recorded for those where its treatment outcome was missing. Please confirm when treatment outcome was recorded (e.g. time to follow-up), and how

Line 141 to 145 “A retrospective cohort study was conducted that included all patients who were notified and diagnosed with MDR/RR-TB, with the start of treatment between January 2013 and December 2015 in Colombia. Data on treatment results were collected until December 2017, if up to this date no record of treatment results existed they were catalogued as missing”.

18. Notification and diagnosis are used interchangeably, helpful to stick to one term only as these can mean slightly different things

We will not indicate at the end of the notification

Statistical Analysis in Methodology

18. **Reconsider the language ‘analyze the interaction’, this does not really reflect the statistical analysis reported in the results – unless you did examine interaction terms? Otherwise suggest ‘to estimate the association between…’**

Line 160 “association between the exposure variables”

19. Please specify which confounders were included in the analysis here, and which (if any) registry variables were excluded

Was answered in question 3 of the major reviews

Definition of Terms

20. Specify what you mean by the ‘Colombian National Policy’

It refers to the guidelines for programmatic management of tuberculosis in Colombia. The phrase was replaced in the text.

Line 191 to 192 “guidelines for management tuberculosis and leprosy program in Colombia”

Results

Descriptive Analysis

21. Figure 2: Is it necessary to show rates of both favourable and unfavourable treatment outcomes on the same graph? Please include y axis for scale. Consider excluding this figure, I am not sure that it helps to answer the study objective.

The figure will be excluded

22. What is the derivation of ‘afro-descendant’? Perhaps consider changing

to Afro-Colombian. Who comprises the ‘other’ group – it seems this is the ‘majority’ – How has ethnicity been defined here?

In Colombia, Afro-descendant is synonymous with Afro-Colombian.

The group called others refers to the mestizos who in Colombia are the majority group

We will change the name to Afro-Colombians and mestizos

23. Reconsider phrasing ‘first’, ‘second’ and ‘third’ care as ‘primary’, ‘secondary’ and ‘tertiary’

The change was made

24. On line 211, please reconsider ‘were affiliated with the subsidized social security and health regime’. Does the subsidised health care also include social protection and other welfare benefits? This contradicts the earlier definition o Treatment outcomes and factors associated with Unfavourable outcomes

Line 244 “were affiliated with the subsidized health regime”

25. Describing ‘the treatment success rate with respect to age showed an inversely proportional trend’ seems like a bit of a leap; could be worth rephrasing, and focussing on the needs of this group more in your discussion

Line 262 to 264 “The older age was associated with a lower success rate, but only patients aged ≥ 60 years showed a significant association with unfavorable outcomes”

26. Table 2: Given you have performed a binomial logistic regression e.g. with a dichotomous outcome variable not multinomial logistic regression, it feels misleading to show success vs. abandonment vs. failure vs. death in Table 2, consider formatting Table 2 with favourable vs. unfavourable outcomes. Please amend 'IC95%' to '95% CI'. Consider rearranging the reference group to first sub-group for every covariate of interest. Please avoid using commas as decimal points. Please include 'Ref.' in your figure legend. Do you think that the 'self-identified ethnicity' variable is limited in its use here?

The suggestion is accepted and table 2 is modified

Discussion

27. 'Notably, the diagnosis and treatment of MDR/RR-TB in Colombia is free for all patients regardless of health regime affiliation' – so actually we are seeing the impact of hidden costs of treatment here? Worth clarifying what is and is not included in your treatment if you are in the subsidised group. Would be helpful to include some background as to catastrophic health spending in Colombia for patients with TB, or more broadly (https://www.researchgate.net/publication/50228851_Determining_factors_of_catastrophic_health_spending_in_Bogota_Colombia)

Line 316 to 320 "Notably, the diagnosis and treatment of MDR/RR-TB in Colombia is free for all patients regardless of health regime affiliation.[14] However there is a differential attention between the two health affiliation regimes, which can be called organizational barrier, which is a factor that hinders the initial contact with the health services (entry barriers) and, also, the timely attention after the patient enters the health center (interior barriers)."

Line 337 to 343 “Also the patients always incur out-of-pocket expenses, which may be direct, such as those related to transportation and examinations or consultations in addition to the provisions of the TB control program, or indirect, such as inability to work due to the disease [24]. These expenses in subsidized families without the ability to pay can create significant barriers to both access and permanence in the TB control program. In this sense, a study carried out in Bogotá, the capital of Colombia, showed that the lower the household income level, the more likely it was to incur catastrophic health spending [25]”.

28. ‘At the national level, incidence and mortality rates, adjusted for age and sex, were higher for the subsidized regime in 37 events of public health interest’ – please clarify what these 37 events are? And how meaningful is this if incidence and mortality has only been adjusted by age and sex.

Line 321 to 328 “Inequalities related to the health system affiliation regime have been identified previously at the national level, for example for the subsidized regime high incidence and mortality rates were identified, adjusted by age and sex, for events tracing the quality of health care, such as mortality in children under five years of age due to acute respiratory infection, acute diarrheal disease, and malnutrition; events related to sexual and reproductive health, such as maternal mortality, gestational syphilis, and congenital syphilis; infectious diseases, such as leishmaniasis, Chagas' disease, and malaria; and poverty-related communicable diseases, such as leprosy and tuberculosis.

29. ‘Other countries such as Nigeria also documented a higher rate of successful TB treatment among patients treated in the private sector’ – however being treated in a public healthcare facility was not independently associated with unfavourable treatment outcome in your multivariate analysis– so I think this comparison may be limited. Are there any other comparable studies in South America?

Suggestion accepted, the comment is removed from the text

30. ‘The care received at first-level HCIs (health centers) was a protective factor for unfavorable outcomes’- Again this did not persist on adjustment in your multivariate model, and it could be worth exploring this further in your discussion

Line 358 to 359 “Care in second and third level health facilities was associated with unfavorable outcomes”

- Conclusions

31. Please consider rephrasing ‘This high rate of unfavourable treatment outcomes was associated with affiliation with the subsidized health system regime and age ≥ 60 years.’ - e.g. could be adapted to: Those who qualified for subsided health care or who

were aged over 60 years were independently more likely to experience unfavourable treatment outcomes

Line 391 to 395 “In conclusion, in Colombia, 50.1% of the patients with RR-TB who initiated treatment between January 2013 and December 2015 showed unfavorable treatment outcomes, and 19.7% died during treatment. Patients with XDR-TB had unfavorable outcomes in 85.7% of cases and with MDR-TB in 47.6% of cases. Patients who qualified for subsidized care or were over 60 years old were more likely to experience unfavorable treatment outcomes independently more likely to experience unfavourable treatment outcomes”

Reviewer #2

1. In page 5 the authors make a comprehensive description of Colombian health system and describe that all TB treatment is provided for free through the Colombian government (TB programme). Nevertheless, later they include a variable that divide health regime in "subsidized" or "contributive". I think the authors maybe should make more clear (if what I understood is correct) that independent of the type of health regime, after being diagnosed, the treatment is provided for free. Also, it would be nice to know if this measure is more likely to a proxy of socioeconomic status than the treatment provision itself.

Modifications were made to clarify this information in both the methods and the discussion: line 121 to 124 "and the subsidized regime, which covers the remaining 53%,[10] sheltering all poor and vulnerable people, for whom health services are covered by government resources.[11] However, people who qualified for subsidized health care services are not necessarily beneficiaries of social protection programs".

Line 136 to 138 "In summary, in Colombia TB is a disease of public health interest. Is of obligatory notification, its diagnosis, treatment and follow-up are covered by public resources"

Line 316 to 320 "Notably, the diagnosis and treatment of MDR/RR-TB in Colombia is free for all patients regardless of health regime affiliation.[14] However there is a differential attention between the two health affiliation regimes, which can be called organizational barrier, which is a factor that hinders the initial contact with the health services (entry barriers) and, also, the timely attention after the patient enters the health center (interior barriers)".

2. In page 6/7, study type and population, please describe the inclusion and exclusion criteria.

Line 141 to 145 “that included all patients who were notified and diagnosed with MDR/RR-TB, with the start of treatment between January 2013 and December 2015 in Colombia. Data on treatment results were collected until December 2017, if up to this date no record of treatment results existed they were catalogued as missing data”.

3. In page 7, row 126/127: the sentence is confusing. Maybe only say “seventy patients with unknown treatment outcome were excluded”? Also, this also should be moved to the results section.

Line 144 to 145 “if up to this date no record of treatment results existed they were excluded, catalogued as missing data”.

Line 224 to 225 “and 70 (13,7%) patients were excluded because treatment outcomes was unknown”

4. In the methods section, it would be important to include a subsection describing the variables that were extracted from the TB dataset and with which purpose.

No variables were extracted, we worked with all the variables available in the database provided by colombia's TB control program

5. In page 7, row 125-133: what method was used to include variables in the adjusted model? Backward, forward or the variables were included all at once? The authors considered any confounding variable.

Line 163 to 165 “Variables with p-values < 0.05 in the bivariate analysis were considered for the multivariate analysis. We did not include or exclude a priori variables from the multivariate analysis”.

6. In page 7, row 125-133: Some variables included in the results section

were not described in the methods. Please include a general description of variables in the adequate section

Line 159 to 162 “Bivariate analysis: A logistic regression model was used to analyze the association between interaction of the exposure variables (age, sex, ethnicity, site of TB, Health regimehealth insurance scheme, treating HCI, level of care, method used for diagnosis and type of resistance) with the response variable (treatment outcome).

7. In page 7, row 125-133: how missing data was handled in the analysis?

Missing data were excluded from the analysis

8. In the methods section, the definition of terms should be included before the statistical analysis.

Suggestion accepted the change was made

9. In page 8, row 171-175: outcomes not evaluated were included or excluded from the analysis? This should be clear in the methods.

Line 141 to 145 “A retrospective cohort study was conducted that included all patients who were notified and diagnosed with MDR/RR-TB, with the start of treatment between January 2013 and December 2015 in Colombia. Data on treatment results were collected until December 2017, if up to this date no record of treatment results existed they were excluded, catalogued as missing data”.

10. In page 9, descriptive analysis, it would be important to describe if there was an increase or decrease in the number of RR-TB over time.

Line 233 to 234 “167 cases were diagnosed in 2013, 181 in 2014 and 163 in 2015”.

11. In page 9, descriptive analysis, the authors could describe the percentage of cases newly diagnosed and those who are being retreated.

Sorry, but we do not have that variable in our database

12. In table 1 and 2, the authors included the diagnosis method as a possible explanatory variable for unfavourable treatment outcomes. What is the hypothesis behind including such a variable?

The diagnostic method of rifampicin resistance was included for a better description of the dynamics of the diagnosis and treatment of RR TB in Colombia; we did not expect it to influence the treatment results. Which was corroborated in the bivariate analysis and did not enter the multivariate analysis.

13. Table 1 is secondary for the authors research question. Maybe it should be moved to supplementary material.

Accepted suggestion

14. In Table 2, please describe which ethnicities were included in the “other” category.

The term others was replaced by Mestizo which is the majority race in Colombia

15. Figure 2 is not very adequate to show what the authors meant. Maybe a simple one bar graph for each city with the proportions of favourable vs unfavourable outcomes and the N on the top would be clearer. Also, if possible, it would be relevant to know the proportion of RR-TB among all TB cases in each city once the N of RR-TB depends on the number of overall TB cases and the population of each city.

This figure was removed from the document at the suggestion of reviewer #1

16. In page 13, row 230: did the authors tested for trend in the relationship between age and lower treatment success?

Line 275 to 277 “older age was associated with a lower success rate, but only patients aged ≥ 60 years showed a significant association with unfavorable outcomes (ORc = 2.4, 95% CI 1.1 - 5.8; ORa = 2.7, 95% CI 1.1 - 6.8)”.

17. In the discussion section, the authors make a very important comparison of the study results with other Colombian literature, but have made a limited comparison with the literature from other LMIC countries or elsewhere. For example, if being treated in the private sector is a proxy of wealth, which other studies have shown an association between wealth and poor treatment outcomes? In the limitation section, absence of further socioeconomic data should be included as a limitation for controlling for confounder in the analysis. There is a vast literature showing the relationship between poverty and unfavourable treatment outcomes, as well the effect of poverty reduction measures on improving TB treatment (PMID: 31000126, PMID: 30740248, PMID: 26884501).

Line 356 to 360 “In Colombia, qualifying to be a beneficiary of the subsidized health regime is synonymous with belonging to the poorest and most vulnerable population, and TB has a direct relationship with poverty and social exclusion. For example, in Brazil, they concluded that being a beneficiary of the Bolsa Familia Program (BFP) was an independent factor that influenced the favorable outcome of people receiving drug treatment for TB”.

18. The paper should be revised by a native English speaker.

Suggestion accepted

American Journal Experts was hired to perform the English translation and style correction of the manuscript

Minor points

1. In the abstract, it is important to make it more clear for the readers what "affiliated with the health regime" means.

Line 121 to 124 "and the subsidized regime, which covers the remaining 53%,[10] sheltering all poor and vulnerable people, for whom health services are covered by government resources.[11] However, people who qualified for subsidized health care services are not necessarily beneficiaries of social protection programs".

2. In page 4, rows 57 – why these conditions represent public health problems?

Line 65 to 69 "These conditions are generally the consequence of social and political decisions that lead to inadequate compliance with the DOTs strategy (strictly supervised shortened treatment) by government entities, sociodemographic barriers that prevent access to medicines (e.g. living in rural areas, not having health insurance"

3. In page 5, row 78 – the word complex does not mean much. The system is complex or the authors meant something else? it would be good to use a more specific word.

Line 94 to 96 "MDR/RR-TB requires prolonged and expensive treatment, which is difficult to sustain in a Colombian health system that requires the joint action of different actors to provide health services"

4. In page 7, row 127: maybe is more accurate to say descriptive analysis instead of frequency analysis?

Line 210 to 211 "A descriptive analysis was performed"

5. In page 7, row 130: A logistic regression model was used to analyze the “association” and not the “interaction” between variables.

Line 212 to 213 “Bivariate analysis: A logistic regression model was used to analyze the association between the exposure variables”

6. In page 8, row148-153: Please describe all the full names before using abbreviations for the first time (eg. RMP, INH and please check in the text.)

Suggestion accepted

Line 62 to 63 “Multidrug resistant tuberculosis (MDR-TB) is defined as TB resistant to at least rifampicin (RMP) and isoniazid (INH), and extensively resistant TB (XDR-TB)”

Thank you for your consideration!

Sincerely,

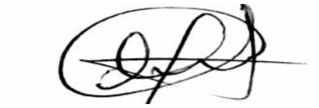

Ninfa Marlen Chaves Torres
Professor, School of Medicine
Nueva Granada Military University
