# Supplementary material for: Factors associated with unfavorable treatment outcomes in patients with rifampicin-resistant tuberculosis in Colombia 2013–2015: A retrospective cohort study
Source: PLoS One. 2021 Apr 14;16(4):e0249565. doi: 10.1371/journal.pone.0249565 (PMC8046199; doi:10.1371/journal.pone.0249565)
Supplement: Attachment — Submitted filename: Cover Letter LC.pdf [file pone.0249565.s004.pdf]

28th October 2020

Dear Dr Tom Wingfield,

I would like to thank you for the opportunity to review this paper entitled ‘Factors associated with unfavourable treatment outcomes in patients with rifampicin-resistant tuberculosis in Colombia 2013-2015: A Retrospective Cohort Study’, and to acknowledge the authors for their careful and thoughtful responses and revisions.

I remain concerned that the English language used in the revised manuscript does not meet the standard required for publication in PLOS ONE. There remain several typographical and grammatical errors throughout the manuscript. Unfortunately, the use of language impacts on the flow of the paper, particularly in the introduction and discussion sections.

Therefore, I advise that this paper requires major revision.

Kind regards,

Dr Louisa Chenciner

BMedSci BMBS MSc (Hons)
