# Supplementary material for: Factors associated with unfavorable treatment outcomes in patients with rifampicin-resistant tuberculosis in Colombia 2013–2015: A retrospective cohort study
Source: PLoS One. 2021 Apr 14;16(4):e0249565. doi: 10.1371/journal.pone.0249565 (PMC8046199; doi:10.1371/journal.pone.0249565)
Supplement: Attachment — Submitted filename: Peer Review .pdf [file pone.0249565.s005.pdf]

Revisions:

Major:

1. Abstract
   Background could be made clearer – and consider avoiding use of jargon ‘health benefit plan administrators’ and ‘control entity’
   Conclusion – suggest rephrasing ‘such outcomes were associated with subsidized health regime affiliation and age > 60 years’; as previously suggested ‘health regime’ is not immediately clear

2. Introduction
   Opening paragraph would benefit from restructuring and more focussed approach to the study context
   Sentence 88-90 remains unclear

3. Methods
   Suggest clearer interpretation of HDI and Gini – as not all readers will immediately understand their significance here
   Are ‘benefit plan administrations’ essentially ‘third party administrators’ – it would be helpful if this terminology is clearer
   Does the National Public Health plan fall under the remit of the state, the health service providers and ‘benefit plan administrations’ or operate independently – I think this could be explained more clearly and succinctly

4. Results
   Table 1 – please specify whether these are row or column % in legend and amend table accordingly and ensure use of decimal points only please (see male p value)
   Please reconsider use of term ‘mestizos’ – does not seem appropriate for use in academic writing

5. Discussion
   Can we be sure that the subsidized health system’s association with unfavourable treatment outcome relates to organisational barriers? Isn’t subsidized healthcare a proxy for social deprivation in your study?
   Out of pocket expenses relating to transport are also considered to be indirect costs – see line 289-291

6. Conclusion
   Remain unconvinced that you can conclude ‘the structure of the Colombian health care model influences MDR-TB treatment results’ based on your results – given there are several missing confounding variables. Consider revision

Minor:

1. Abstract
   Methods – specify type of logistic regression model e.g., multivariate
   Results – aim for consistency in formatting e.g. n =; %; suggest 'revealed' is too informal for academic writing

2. Introduction
   Suggest use of 'adherence' as opposed to 'compliance', and ensure consistently used
   Clarify whether WHO estimates on line 70-73 are globally
   Suggest restructuring of sentence 81-85 e.g. 'Different factors, including education, race, age etc. are associated with higher likelihood of unfavourable outcome'
   Suggest use of 'treatment outcomes' as opposed to 'results of treatment' for consistency
   Are there studies which you could cite here – are they mostly focussed on the capital? -line 87
   Suggest on line 94 – instead of 'thus decrease' consider 'may contribute to reduction in unfavourable treatment outcome' – treatment failure is slightly different

3. Methods
   Suggest avoiding use of term 'developing countries'
   Consider rephrasing line 134-135 'trusting that most patients would complete treatment'
   Suggest specifying this data is anonymised or depersonalised on line 138
   Reconsider use of language in paragraph line 165-168 – use of term 'abandonment' and 'treatment was finished'
   Line 188 suggest specifying 'a logistic regression model'

4. Results
   Ensure consistency in how results are reported (n = X; %)
   Suggest rephrasing line 239 'significant association with'

7. Discussion
   Avoid use of 'i.e' in academic writing if able – see line 275
   Consider rephrasing sentence 291-293
   Consider rephrasing 299 'the outcome of drug treatment for TB was favourable for beneficiaries of the BFP' – implies this was the case for all BFP recipients
   What do you mean by 'length of stay in the TB control program'?
   Line 333 – please clarify what is meant by 'health status' here?
   Suggest shortening sentence line 335-338
   Suggest 'likely representative' is used on line 338
