# Supplementary material for: Factors associated with unfavorable treatment outcomes in patients with rifampicin-resistant tuberculosis in Colombia 2013–2015: A retrospective cohort study
Source: PLoS One. 2021 Apr 14;16(4):e0249565. doi: 10.1371/journal.pone.0249565 (PMC8046199; doi:10.1371/journal.pone.0249565)
Supplement: Attachment — Submitted filename: Response to Reviewers.pdf [file pone.0249565.s006.pdf]

Dr. Olivier Neyrolles

Academic Editor

PLOS ONE

Response to Reviewers

Ref: PONE-D-20-18648

Factors associated with unfavorable treatment outcomes in patients with rifampicin-resistant tuberculosis in Colombia

Dear Dr. Olivier Neyrolles

Below, we respond to questions and suggestions from the editor and reviewers. We hope to meet the requirements and expectations of PLOS ONE. We are available for further questions or suggestions about this paper.

Yours sincerely,

Marlen Chaves

Revisions:

Major:

1. Abstract: Background could be made clearer – and consider avoiding use of jargon 'health benefit plan administrators' and 'control entity' Conclusion – suggest rephrasing 'such outcomes were associated with subsidized health regime affiliation and age > 60 years'; as previously suggested 'health regime' is not immediately clear

Minor:

1. Abstract Methods – specify type of logistic regression model e.g., multivariate Results – aim for consistency in formatting e.g. n =; %; suggest 'revealed' is too informal for academic writing

"Background: Multidrug-resistant rifampicin-resistant tuberculosis (MDR/RR-TB) requires prolonged and costly treatment, which is difficult to sustain in the Colombian health system. This requires the joint action of the Health Promoting Companies and health service providers, under the direction, regulation, supervision, monitoring and control of the national government and the Ministry of Health, to provide timely health services to people with TB. The identification of factors associated with unfavorable treatment outcomes in MDR/RR TB patients who received drug therapy between 2013 and 2015 in Colombia can help guide the design of national public health strategies.

Method: A retrospective cohort study was conducted with all patients who received treatment for MDR/RR-TB between January 2013 and December 2015 in Colombia and were registered and followed up by the national TB control program. A multivariate logistic regression model was used to analyze the associations between the exposure variables with the response variable (treatment outcome).

Results: A total of 511 patients with MDR/RR-TB were registered and followed up by the national TB control program in Colombia, of whom 16 (3.1%) had extensive drug resistance, 364 (71.2%) had multidrug resistance, and 131 (25.6%) had RMP monoresistance. The mean age was 39.9 years (95% confidence interval (CI): 38.5-41.3), most patients were male 285 (64.6%), and 299 (67.8%) people qualified for subsidized health care services. The rate of unfavorable treatment outcomes in the RR-TB cohort was 50.1%, with rates of 85.7% for patients with extensive drug resistance, 47.6% for patients with multidrug resistance, and 52.6% for patients with RMP monoresistance. The 511 MDR/RR-TB patients were included in bivariate and multivariate analyses, patients age ≥ 60 years (crude odds ratio (ORc) = 2.4, 95% CI 1.1 – 5.8; adjusted odds ratio (ORa) = 2.7, 95% CI 1.1 – 6.8) and subsidized health regime affiliation (ORc = 3.6, 95% CI 2.3 – 5.6; ORa = 3.4, 95% CI 2.0 – 6.0) were associated with unfavorable treatment outcomes.

Conclusion: More than 50% of the patients with MDR/RR-TB in Colombia experienced unfavorable treatment outcomes, and the patients who were eligible for subsidized care or were older than 60 years were more likely to experience unfavorable treatment outcomes."

Major:

2. Introduction Opening paragraph would benefit from restructuring and more focussed approach to the study context

"Represents a threat to the control of TB because its treatment is more expensive, more toxic and its prognosis is much worse than that of those infected by sensitive strains. Non-adherence to treatment and non-implementation of the directly observed treatment (DOT) strategy have been associated with the development of MDR/RR-TB. In addition other factors, such as sociodemographic barriers that impede access to medicines (e.g., living in rural areas, not having health insurance)."

Sentence 88-90 remains unclear

"Also the proportion of unfavorable treatment outcomes in MDR/RR-TB is higher than that in TB with drug-sensitive bacilli."

Minor:

Suggest use of 'adherence' as opposed to 'compliance'

"Non-adherence to treatment and non-implementation of the directly observed treatment (DOT) strategy have been associated with the development of MDR/RR-TB."

and ensure consistently used Clarify whether WHO estimates on line 70-73 are globally

"The World Health Organization (WHO) estimated 484,000 cases (range, 417,000-556,000) of MDR/RMP-resistant (RR)-TB globally in 2018 compared to 160,684 cases in 2017"

Suggest restructuring of sentence 81-85 e.g. 'Different factors, including education, race, age etc. are associated with higher likelihood of unfavourable outcome' Suggest use of 'treatment outcomes' as opposed to 'results of treatment' for consistency

"Different factors, including education, race, age, drug use, history of second-line treatment, resistance to fluoroquinolones, positive sputum smear after two months of treatment, and XDR-TB are associated with higher likelihood of unfavourable

outcome. [4,5,6], and coinfection with HIV has been specifically associated with death[5].”

Are there studies which you could cite here – are they mostly focussed on the capital?

“Studies on the treatment outcomes results of treatment for MDR/RR-TB in Colombia are scarce and located in some cities that are not representative of the Colombian population”

-line 87 Suggest on line 94 – instead of ‘thus decrease’ consider ‘may contribute to reduction in unfavourable treatment outcome’ – treatment failure is slightly different

“may help guide the design of national public health strategies aimed at improving the care provided to patients with MDR/RR-TB may contribute to reduction in unfavourable treatment outcome”

Major:

3. Methods suggest clearer interpretation of HDI and Gini – as not all readers will immediately understand their significance here

“Although it has managed to surpass the minimum achievements, placing it in the high human development category (Human Development Index (HDI) of 0.761). There is a poor redistribution of income that perpetuates inequality.”

Are ‘benefit plan administrations’ essentially ‘third party administrators’ – it would be helpful if this terminology is clearer Does the National Public Health plan fall under the remit of the state, the health service providers and ‘benefit plan administrations’ or operate independently – I think this could be explained more clearly and succinctly

“This structure also includes the National Public Health Plan, which is incumbent upon the state”

Minor

3. Methods Suggest avoiding use of term ‘developing countries’

Accepted suggestion the term is eliminated.

Consider rephrasing line 134-135 ‘trusting that most patients would complete treatment’

“Data for treatment results were collected until December 2017 assuming a period of at least two years bearing in mind that in these cases the total treatment duration should be at least 20 months”

Suggest specifying this data is anonymised or depersonalised on line 138

“The Ministry of Health and Social Protection of Colombia (MINSALUD) provided the participants’ data in a database with individualized and anonymised information.”

Reconsider use of language in paragraph line 165-168 – use of term ‘abandonment’ and ‘treatment was finished’

“Treatment was favorable when the patient was considered cured or finished treatment. An unfavorable outcome was considered when the result was treatment abandonment, treatment failure, or death.”

Line 188 suggest specifying ‘a logistic regression model’

“A multivariate logistic regression model was used”

4. Results Table 1 – please specify whether these are row or column % in legend and amend table accordingly and ensure use of decimal points only please (see male p value) Please reconsider use of term ‘mestizos’ – does not seem appropriate for use in academic writing

Suggestions were accepted, corrections were made to the table.

Minor

4. Results Ensure consistency in how results are reported (n = X; %)

Suggestions were accepted

Suggest rephrasing line 239 ‘significant association with’

“Age ≥ 60 years showed association with unfavorable outcomes”

Major:

5. Discussion Can we be sure that the subsidized health system’s association with unfavourable treatment outcome relates to organisational barriers? Isn’t subsidized healthcare a proxy for social deprivation in your study?

“and we speculate that in addition to the social and economic deprivation to which the population of the subsidized regime may be exposed, there are differences in the care provided to patients between the two health affiliation regimes”

Out of pocket expenses relating to transport are also considered to be indirect costs – see line 289-291

“In addition, patients incur out-of-pocket expenses, which may be direct, such as those related to transportation and examinations or consultations or indirect, such as an inability to work due to the disease”

Minor:

7. Discussion Avoid use of 'i.e' in academic writing if able – see line 275

i.e eliminated

Consider rephrasing sentence 291-293

"These expenses in in low-income families can create significant barriers to both access to the TB control program and compliance with TB treatment."

Consider rephrasing 299 'the outcome of drug treatment for TB was favourable for beneficiaries of the BFP' – implies this was the case for all BFP recipients

"This relationship between poverty and TB was studied in Brazil, where the authors concluded that TB patients who were beneficiaries of a government cash grant, in this case the Bolsa Familia Program (PBF), obtained favorable results in the TB treatment."

What do you mean by 'length of stay in the TB control program'?

"and the patient's follow-up time in the tuberculosis control program."

Line 333 – please clarify what is meant by 'health status' here?

"The absence of these variables may lead to overestimation or underestimation of the association between health status and TB treatment outcomes in this cohort of patients."

Suggest shortening sentence line 335-338

"The missing data were 70/511, representing a loss of 13.7%, which is acceptable, and the missing cases did not have different characteristics compared to those included in the analysis"

Suggest 'likely representative' is used on line 338

"the results obtained are likely representative of the Colombian population"

6. Conclusion Remain unconvinced that you can conclude 'the structure of the Colombian health care model influences MDR-TB treatment results' based on your results – given there are several missing confounding variables. Consider revision

"Our results suggest that in addition to the conditions of vulnerability to which the Colombian population affiliated to the subsidized health care system may be exposed, the structure of the Colombian health care model may influence MDR-TB treatment outcomes, which should be considered in the design of public health policies."
