# Supplementary material for: Factors associated with unfavorable treatment outcomes in patients with rifampicin-resistant tuberculosis in Colombia 2013–2015: A retrospective cohort study
Source: PLoS One. 2021 Apr 14;16(4):e0249565. doi: 10.1371/journal.pone.0249565 (PMC8046199; doi:10.1371/journal.pone.0249565)
Supplement: Attachment — Submitted filename: Manuscript Revisions February 2021.pdf [file pone.0249565.s007.pdf]

Minor Revisions

Abstract

Line 28-29: Unclear what 'Health Promoting Enterprises' are, and unclear why capitalised. Suggest avoiding using jargon here – and just reference joint action of different service providers

Lines 28-31: Suggest condensing this sentence

Line 34: 'National public health strategies' or national TB control programmes? Suggest keeping it specific and focussed

Line 37: suggest substituting 'and were registered' with 'who were registered'

Line 38 and Line 193: suggest substituting 'analyze' with 'estimate'

Line 44: suggest omitting 'people' in '299 (67.8%) people qualified'

Line 54 -55: suggest separating into two separate sentences for clarity e.g. '..unfavourable treatment outcomes [FULL STOP] Patients who were eligible for subsidized care or over 60 years of age were more likely to experience unfavourable treatment outcomes.'

Introduction

Line 68: Please revise this sentence – suggest 'MDR/RR-TB poses a threat to TB control – patients have worse (long term?) prognosis, and treatment is more likely to be expensive and present toxicity'

Line 70-71: Suggest 'lack of treatment support (including DOT) and limited treatment adherence have both been associated with the development of MDR/RR-TB'

Line 72-73: Ensure this sentence makes grammatical sense – suggest 'Other contributing factors include social and cultural barriers to access, such as living in rural areas and the absence of (private?) health insurance'

Line 82-84: 'Also' not necessary here – Consider 'The proportion of patients experiencing unfavourable treatment outcome is higher in MDR/RR-TB than in TB with drug sensitive bacilli'

Line 84: Suggest 'Other factors' not 'Different factors'

Line 90: Consider cutting 'additionally' and cutting 'requires the joint action of different actors within the same system to provide health services' instead 'MDR/RR-TB often necessitates prolonged and more expensive treatment, which is challenging to sustain in the current Colombian health care system'

Line 93-97: Suggest condensing to 'Identifying factors associated with unfavourable treatment outcome for patients with MDR/RR-TB in Colombia, may support design and delivery of the national TB control programmes'

Line 99: Suggest 'treatment' not 'pharmacotherapy' for consistency please

Methods

Line 103: Please omit 'it has managed to surpass the minimum achievements' – suggest 'Colombia is an upper-middle income country (Human Development Index 0.761), however there remains significant wealth inequality'

Line 106: Suggest omitting 'the social security system for health care' and instead 'the public health system encounters…'

Line 109-112: Suggest separating into two sentences e.g. '…95% of the population [STOP]. This organisation separates insurance and administration of financial resources, from the (direct?) service provision management of its members' – still not fully clear what service provision management entails

Line 117-118: Consider 'who rely on public healthcare services' instead of 'for whom health services are covered by government resources'

Line 118: use 'qualifying' not 'qualified'

Line 121: apologies – still unclear what 'Health Promoting Enterprises' are – and I wonder if you are referencing 'Health Solidarity Enterprises' (Empresas Solidarias de Salud, ESSs) instead? https://jech.bmj.com/content/jech/56/10/742.full.pdf - please clarify

Line 124: Please condense 'including healthcare institutions, such as..' to 'including hospitals and community clinics'

Line 126: Suggest condensing 'The Colombian Government's National Public Health Plan prioritises national health promotion and disease prevention, including infectious diseases such as TB, Leprosy and Malaria. Therefore, TB is a disease of public health interest, and its diagnosis, treatment and follow-up are publicly funded as a result'

Line 135: Grammar – replace 'received' with 'receiving'

Line 136: Suggest 'treatment outcomes' in lieu of 'treatment results' for consistency

Line 136-138: Grammar – more than one clause in this sentence – consider revision – e.g., appropriate punctuation

Line 140: Suggest revision of 'their information was catalogued as missing data' – if they were excluded then why were they categorised as missing?

Line 141-142: 'individualised and anonymised information' – please clarify which it is – this is contradictory!

Line 169-170: 'favourable treatment or unfavourable outcome' – for purposes of consistency please either refer to treatment outcome

Line 172 and line 183: As previously mentioned – please avoid stigmatising language – 'treatment abandonment'

Line 190-191: Again, not entirely clear were these patients excluded through the study inclusion criteria in which case no need to mention again here

Results

Line 217: Again, shouldn't the 70 (13.7%) be included in those who did not meet your inclusion criteria?

Line 230: Please format (n = ; %)

Line 233: Suggest omitting 'nine of which were capitals'

Line 238-239: Suggest two separate sentences e.g. '…RR-TB showed unfavourable treatment outcomes. [STOP] 122 (27.7%)'

Discussion

Line 268-269: Suggest two separate sentences and condense 'was found in this MDR/RR-TB cohort. [STOP]' 47.4% of patients with monoresistance to RMP experienced favourable outcome…'

Line 279: Again, please keep language consistent TB treatment outcomes, not 'results of TB treatment'

Line 280: Suggest two separate sentences 'is relevant. [STOP] Individuals affiliated with…'

Line 283-289: This sentence needs to be split into two/three – important discussion, but can be made clearer through restructuring.

Line 290: suggest 'a national level' not 'the national level'

Line 290-294 suggest condensing 'for patients receiving care in the subsidised healthcare system, higher under-5 mortality, maternal mortality and mortality related to communicable disease has already been observed'

Line 298 Please avoid stigmatising language – suggest 'adherence' not 'compliance'

Line 298-300: I think you could relate this more closely to your results to strengthen your argument!

Line 305-306: suggest 'were more likely to experience favourable outcome' not 'obtained favourable results'

Line 331-332: suggest omitting 'thus all available variables were analyzed'

Line 332: avoid use of 'influence' – this implies extent of temporal relationship

Line 333: what do you mean by 'exclude confounding factors' – I'm not sure your analysis is able to achieve this

Line 338: please be more specific about the nature of bias this may introduce and the effect on your main findings

Line 340: please avoid use of fractions interspersed in text e.g. '70/511'

Line 346: what do you mean by 'terms of updating' consider rephrasing

Line 355-356: Suggest keeping conclusion more specific e.g. design of TB control programmes, or strategies to reduce burden of MDR/RR-TB
