# Supplementary material for: Factors associated with unfavorable treatment outcomes in patients with rifampicin-resistant tuberculosis in Colombia 2013–2015: A retrospective cohort study
Source: PLoS One. 2021 Apr 14;16(4):e0249565. doi: 10.1371/journal.pone.0249565 (PMC8046199; doi:10.1371/journal.pone.0249565)
Supplement: Attachment — Submitted filename: Response to Reviewers.docx [file pone.0249565.s008.docx]

**Dr. Olivier Neyrolles**
Academic Editor
PLOS ONE

Response to Reviewers

Ref: PONE-D-20-18648
Factors associated with unfavorable treatment outcomes in patients with rifampicin-resistant tuberculosis in Colombia

Dear Dr. Olivier Neyrolles

Below, we respond to questions and suggestions from the editor and reviewers. We hope to meet the requirements and expectations of PLOS ONE. We are available for further questions or suggestions about this paper.

Yours sincerely,

Marlen Chaves

**Revisions:**

**Minor Revisions**

**Abstract**

Line 28-29: Unclear what ‘Health Promoting Enterprises’ are, and unclear why capitalised. Suggest avoiding using jargon here – and just reference joint action of different service providers

“This requires the joint action of different providers to provide timely health services to people with TB”

Lines 28-31: Suggest condensing this sentence

“This requires the joint action of different providers to provide timely health services to people with TB”

Line 34: ‘National public health strategies’ or national TB control programmes? Suggest keeping it specific and focussed

“Identifying factors associated with unfavorable treatment outcomes in patients with MDR/RR-TB who received drug therapy between 2013 and 2015 in Colombia can help guide the strengthening of the national TB control program.”

Line 37: suggest substituting ‘and were registered’ with ‘who were registered’

“who were registered and were registered and followed up by the national TB control program”

Line 38 and Line 193: suggest substituting ‘analyze’ with ‘estimate’

“was used to estimate the associations between the exposure variables with the response variable (treatment outcome)”

Line 44: suggest omitting ‘people’ in ‘299 (67.8%) people qualified’

“were eligible for subsidized health services”

Line 54 -55: suggest separating into two separate sentences for clarity e.g. ‘..unfavourable treatment outcomes [FULL STOP] Patients who were eligible for subsidized care or over 60 years of age were more likely to experience unfavourable treatment outcomes.’

“The patients who were eligible for subsidized care were more likely to experience unfavorable treatment outcomes. Those who were older than 60 years were also more likely to experience unfavorable treatment outcomes”

**Introduction**

Line 68: Please revise this sentence – suggest ‘MDR/RR-TB poses a threat to TB control – patients have worse (long term?) prognosis, and treatment is more likely to be expensive and present toxicity’

Suggestion accepted, it was corrected in document

Line 70-71: Suggest ‘lack of treatment support (including DOT) and limited treatment adherence have both been associated with the development of MDR/RR-TB’

Suggestion accepted, it was corrected in document

Line 72-73: Ensure this sentence makes grammatical sense – suggest ‘Other contributing factors include social and cultural barriers to access, such as living in rural areas and the absence of (private?) health insurance’

Suggestion accepted, it was corrected in document

Line 82-84: ‘Also’ not necessary here – Consider ‘The proportion of patients experiencing unfavourable treatment outcome is higher in MDR/RR-TB than in TB with drug sensitive bacilli’

Suggestion accepted, it was corrected in document

Line 84: Suggest ‘Other factors’ not ‘Different factors’

Suggestion accepted, it was corrected in document

Line 90: Consider cutting ‘additionally’ and cutting ‘requires the joint action of different actors within the same system to provide health services’ instead ‘MDR/RR-TB often necessitates prolonged and more expensive treatment, which is challenging to sustain in the current Colombian health care system’

Suggestion accepted, it was corrected in document

Line 93-97: Suggest condensing to ‘Identifying factors associated with unfavourable treatment outcome for patients with MDR/RR-TB in Colombia, may support design and delivery of the national TB control programmes’

Suggestion accepted, it was corrected in document

Line 99: Suggest ‘treatment’ not ‘pharmacotherapy’ for consistency please

Suggestion accepted, it was corrected in document

**Methods**

Line 103: Please omit ‘it has managed to surpass the minimum achievements’ – suggest ‘Colombia is an upper-middle income country (Human Development Index 0.761), however there remains significant wealth inequality’

Suggestion accepted, it was corrected in document

Line 106: Suggest omitting ‘the social security system for health care’ and instead ‘the public health system encounters…’

Suggestion accepted, it was corrected in document

Line 109-112: Suggest separating into two sentences e.g. ‘…95% of the population [STOP]. This organization separates insurance and administration of financial resources, from the (direct?) service provision management of its members’ – still not fully clear what service provision management entails

Suggestion accepted, it was corrected in document

Line 117-118: Consider ‘who rely on public healthcare services’ instead of ‘for whom health services are covered by government resources’

Suggestion accepted, it was corrected in document

Line 118: use ‘qualifying’ not ‘qualified’

Suggestion accepted, it was corrected in document

Line 121: apologies – still unclear what ‘Health Promoting Enterprises’ are – and I wonder if you are referencing ‘Health Solidarity Enterprises’ (Empresas Solidarias de Salud, ESSs) instead? https://jech.bmj.com/content/jech/56/10/742.full.pdf -

Are not "solidarity health companies". Health insurance companies are private health insurance companies. <https://doi.org/10.1080/09581596.2018.1449943>

Please clarify Line 124: Please condense ‘including healthcare institutions, such as..’ to ‘including hospitals and community clinics’

Suggestion accepted, it was corrected in document

Line 126: Suggest condensing ‘The Colombian Government’s National Public Health Plan prioritises national health promotion and disease prevention, including infectious diseases such as TB, Leprosy and Malaria. Therefore, TB is a disease of public health interest, and its diagnosis, treatment and follow-up are publicly funded as a result’

Suggestion accepted, it was corrected in document

Line 135: Grammar – replace ‘received’ with ‘receiving’

Ok

Line 136: Suggest ‘treatment outcomes’ in lieu of ‘treatment results’ for consistency

Ok

Line 136-138: Grammar – more than one clause in this sentence – consider revision – e.g., appropriate punctuation

“Data for treatment outcomes were collected until December 2017, assuming a period of at least two years bearing in mind that in these cases the total treatment duration should be at least 20 months”

Line 140: Suggest revision of ‘their information was catalogued as missing data’ – if they were excluded then why were they categorised as missing?

Line 141-142: ‘individualised and anonymised information’ – please clarify which it is – this is contradictory!

“Patients were excluded if no record of treatment results up to this date was available, and their information was catalogued as missing data. The Ministry of Health and Social Protection of Colombia (MINSALUD) provided the participants’ data in a database with individualized and anonymised information”

Line 169-170: ‘favourable treatment or unfavourable outcome’ – for purposes of consistency please either refer to treatment outcome. Ok

Line 172 and line 183: As previously mentioned – please avoid stigmatising language – ‘treatment abandonment’

Was changed to “lost to follow up”

Line 190-191: Again, not entirely clear were these patients excluded through the study inclusion criteria in which case no need to mention again here

the phrase “Seventy patients categorized as not evaluated were not included in the analysis because the treatment outcome was unknown” was removed from the document

**Results**

Line 217: Again, shouldn’t the 70 (13.7%) be included in those who did not meet your inclusion criteria?

the phrase “and 70 (13.7%) patients were excluded because their treatment outcomes were unknown” was removed from the document

Line 230: Please format (n = ; %)

“most patients were male (n=285; 64.6%)”

Line 233: Suggest omitting ‘nine of which were capitals’

Suggestion accepted, it was corrected in document

Line 238-239: Suggest two separate sentences e.g. ‘…RR-TB showed unfavourable treatment outcomes. [STOP] 122 (27.7%)’

Suggestion accepted, it was corrected in document

**Discussion**

Line 268-269: Suggest two separate sentences and condense ‘was found in this MDR/RR-TB cohort. [STOP]’ 47.4% of patients with monoresistance to RMP experienced favourable outcome…’

“An overall therapeutic success rate of 49.9% was found in this MDR/RR-TB cohort. 47.4% among patients with monoresistance to RMP, 52.4% among patients with MDR-TB, and 18.6% among patients with XDR-TB.”

Line 279: Again, please keep language consistent TB treatment outcomes, not ‘results of TB treatment’

“The association between the health regime and the TB treatment outcomes”

Line 280: Suggest two separate sentences ‘is relevant. [STOP] Individuals affiliated with…’

Suggestion accepted, it was corrected in document

Line 283-289: This sentence needs to be split into two/three – important discussion, but can be made clearer through restructuring.

“Although the diagnosis and treatment of MDR/RR-TB in Colombia are free for all patients regardless of their health regime affiliation, discrepancies remain in the outcomes of treatment. We speculate that in addition to the social and economic deprivation to which the population of the subsidized regime may be exposed, there are differences in the care provided to patients between the two health affiliation regimes. Which can be considered an organizational barrier, a factor hindering initial contact with health services (entry barriers) and timely care after a patient enters the health center (interior barriers)”

Line 290: suggest ‘a national level’ not ‘the national level’

Suggestion accepted, it was corrected in document

Line 290-294 suggest condensing ‘for patients receiving care in the subsidised healthcare system, higher under-5 mortality, maternal mortality and mortality related to communicable disease has already been observed’

Suggestion accepted, it was corrected in document

Line 298 Please avoid stigmatising language – suggest ‘adherence’ not ‘compliance’

Suggestion accepted, it was corrected in document

Line 298-300: I think you could relate this more closely to your results to strengthen your argument!

“These expenses in in low-income families (As is the case for families in the subsidized regime) can create significant barriers to both access to the TB control program and adherence with TB treatment”

Line 305-306: suggest ‘were more likely to experience favourable outcome’ not ‘obtained favourable results’

Suggestion accepted, it was corrected in document

Line 331-332: suggest omitting ‘thus all available variables were analyzed’

Suggestion accepted, it was corrected in document

Line 332: avoid use of ‘influence’ – this implies extent of temporal relationship

Line 333: what do you mean by ‘exclude confounding factors’ – I’m not sure your analysis is able to achieve this

Suggestion accepted, it was corrected in document

Line 338: please be more specific about the nature of bias this may introduce and the effect on your main findings

“Due to a selection bias, which prevents extrapolating our results to the Colombian population with any of the aforementioned conditions”

Line 340: please avoid use of fractions interspersed in text e.g. ‘70/511’

Suggestion accepted, it was corrected in document

Line 346: what do you mean by ‘terms of updating’ consider rephrasing

“it can still be improved the quality of the recorded data to reduce lost data and facilitate collection of the largest number of variables possible in reports for researchers who request these data”

Line 355-356: Suggest keeping conclusion more specific e.g. design of TB control programmes, or strategies to reduce burden of MDR/RR-TB
